# Lymph node metastatic patterns and the development of multidisciplinary treatment for esophageal cancer

**DOI:** 10.1093/dote/doad006

**Published:** 2023-03-01

**Authors:** Satoru Matsuda, Masashi Takeuchi, Hirofumi Kawakubo, Yuko Kitagawa

**Affiliations:** Department of Surgery, Keio University School of Medicine, Tokyo, Japan; Department of Surgery, Keio University School of Medicine, Tokyo, Japan; Department of Surgery, Keio University School of Medicine, Tokyo, Japan; Department of Surgery, Keio University School of Medicine, Tokyo, Japan

**Keywords:** esophageal cancer, lymph node metastasis, multidisciplinary treatment, three-field lymph adenectomy

## Abstract

Abundant lymphatic flow and the anatomical location of the esophagus can result in the widespread distribution of lymph node metastasis of esophageal cancer from the cervical to the abdominal field. Historically, the Japan Esophageal Society and American Joint Committee on Cancer offer two different classifications of lymph node group location surrounding the esophagus. The location of sentinel lymph nodes in midthoracic esophageal cancer reflects the variety of lymphatic drainage routes. In fact, in cT1N0 esophageal cancer, pathological lymph node metastasis has been observed from the cervical to the abdominal field, and the locations were shown to be closely linked to the primary tumor location in advanced stages. While the impact of histology on the distribution of LN metastasis has been extensively debated, a recent prospective study on esophagogastric junction cancer found that metastatic patterns did not differ by histology. Thoracic duct lymph nodes were defined as one of the regional lymph node stations in the mediastinum. Although lymph node metastasis around the thoracic duct has occasionally been observed, the oncologic impact of thoracic duct lymph node dissection has not been fully elucidated. To eradicate tumors locoregionally, three-field lymph node dissection, a strategy for extended lymph node clearance, has been established. In esophagectomy, three-field lymph node dissection is defined as a procedure for complete regional cervico-thoraco-abdominal lymph node dissection. However, its therapeutic efficacy must be evaluated based on the balance between oncological outcomes and possible added surgical risk. To further improve survival, multidisciplinary treatment consisting of surgery, chemotherapy, and radiotherapy has been established worldwide as a standard treatment for esophageal cancer. Now that neoadjuvant therapy followed by esophagectomy is the standard, adding adjuvant therapy including immunotherapy could be a promising treatment option. The ideal combination of various multidisciplinary treatment approaches and extensive LN dissection need to be established to improve the oncological outcomes for EC patients.

## INTRODUCTION

Esophagectomy has long been used as a curative treatment for esophageal cancer (EC). Lymph node (LN) metastasis is a major reason for the poor prognosis of EC patients. EC can metastasize even in the early stage disease due to its anatomical location and histological characteristics, such as an abundant lymph–capillary network. Furthermore, the distribution of LN metastasis can vary depending on clinicopathological factors such as primary tumor and depth of invasion. The distribution of LN and its metastatic patterns has been debated due to significant differences in clinicopathological factors and standard treatment across countries. We discussed the LN metastatic patterns and the development of multidisciplinary treatment for EC in this review.

## EPIDEMIOLOGY

EC is a common cancer that ranks seventh in terms of incidence and sixth in terms of death.^1^ Esophageal squamous cell carcinoma (ESCC) is predominant in East Asia and Africa, whereas esophageal adenocarcinoma (EAC) represents two-thirds of EC cases in Europe and North America. Despite advances in treatment, the prognosis of EC still largely depends on disease progression. According to the 2014 comprehensive registry by the Japan Esophageal Society (JES), the 5 year survival rate of patients with EC who have undergone esophagectomy is 82.4% for those with cStage IA, 62.6% for IB, 52.0% for IIA, 67.5% for IIB, 48.1% for IIIA, 44.3% for IIIB, 39/1% for IIIC, and 35.4% for IV.^2^

## LN METASTATIC PATTERNS FOR EC

### Classification of LN stations in EC

Historically, there are two different classifications for group location of LNs surrounding the esophagus. One is produced by the JES^3^^,^^4^ and the other by the American Joint Committee on Cancer (AJCC).^5^ Both classifications are matched and described in Table 1, which was modified from our proposal for uniformity in classification of LN stations in EC.^6^ Almost all stations can be classified as independent. Although both the JES and AJCC classifications can be used to name each LN station, the adequate field of LN dissection should be considered based on the safety and efficacy of lymphadenectomy. Thus, the recommended field of LN dissection can be described in the treatment guidelines.^7^^,^^8^ To develop uniformed criteria and a consensus field of lymphadenectomy, a prospective observational study, the TIGER study, is being conducted.^9^

**Table 1:** JES and AJCC classification for lymph node stations in EC

JES (11th)		AJCC (8th)
Cervical LNs		
101 (L/R)	Cervical paraesophageal LNs	1 R/L (IV^†^)
102up	Upper deep cervical LNs	IIB^†^
102 mid	Middle deep cervical LNs	III^†^
103	Peripharyngeal LNs	IIA and III^†^
104 (L/R)	Supraclavicular LNs	IV and VB^†^
Thoracic LNs		
105	Upper thoracic paraesophageal LNs	8up
106recL	Left recurrent nerve LNs	2 L
106recR	Right recurrent nerve LNs	2 R
106pre	Pretracheal LNs	4 R
106tbL	Left tracheobronchial LNs	4 L
106tbR	Right tracheobronchial LNs	4 R
107	Subcarinal LNs	7
108	Middle thoracic paraesophageal LNs	8 m
109 L	Left main bronchus LNs	10^‡^
109R	Right main bronchus LNs	10^‡^
110	Lower thoracic paraesophageal LNs	8lo
111	Supradiaphragmatic LNs	15
112aoA	Anterior thoracic paraaortic LNs	8 m and 8lo
112aoP	Posterior thoracic paraaortic LNs	8 m and 8lo
112pul (L/R)	Pulmonary ligament LNs	9 R/L
113	Ligamentum arteriosum LNs (Botallo LNs)	5
Abdominal LNs		
1	Right paracardial LNs	16
2	Left paracardial LNs	16
3a	Lesser curvature LNs along the branches of the left gastric artery	17
7	LNs along the left gastric artery	17
8a	LNs along the common hepatic artery (anterosuperior group)	18
9	LNs along the celiac artery	20
11p	LNs along the proximal splenic artery	19
11d	LNs along the distal splenic artery	19
19	Infradiaphragmatic LNs	16
20	LNs in the esophageal hiatus of the diaphragm	16

AJCC, American Joint Committee on Cancer; EC, esophageal cancer; JES, Japan Esophageal Society; LN, lymph node.

^†^AJCC head and neck cancer staging (eighth edition).

^‡^AJCC lung cancer staging (eighth edition).

### Mapping of sentinel LN and metastatic LNs in the early stage

**Fig. 1:**
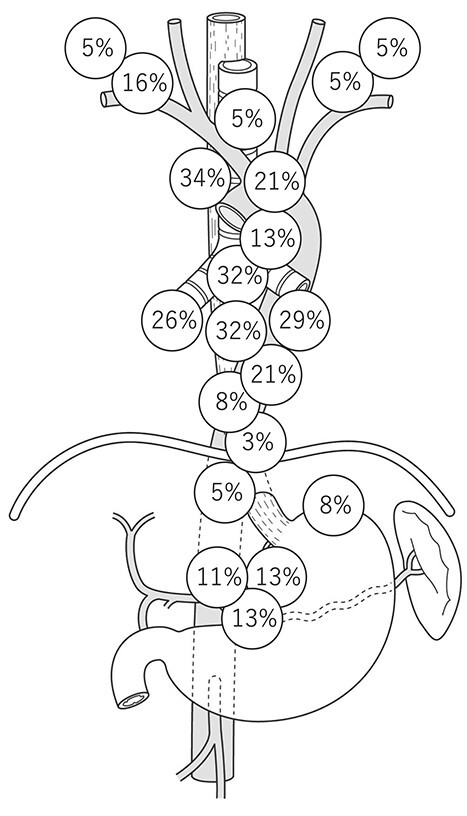
Distribution of sentinel LNs of cT1b/2N0 EC located at the midthoracic esophagus. (EC, esophageal cancer; LN, lymph node.)

**Fig. 2:**
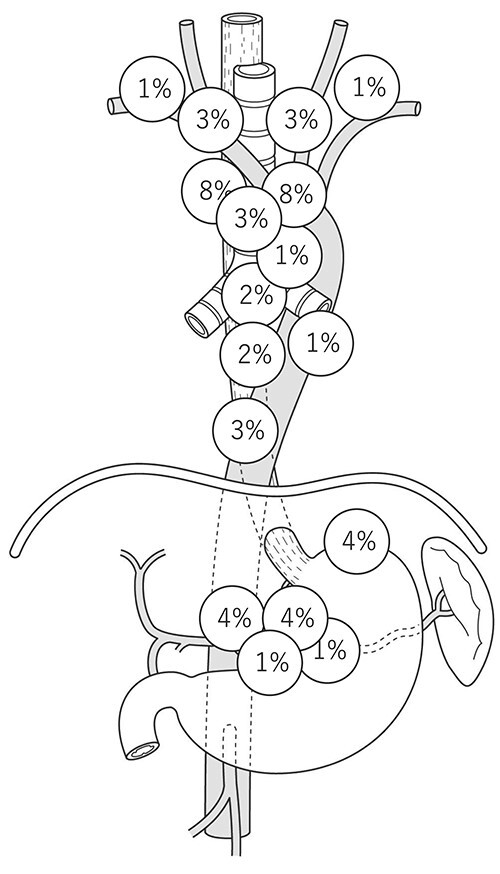
Distribution of metastatic LNs in cT1bN0 EC located at the midthoracic esophagus. (EC, esophageal cancer; LN, lymph node.)

**Fig. 3:**
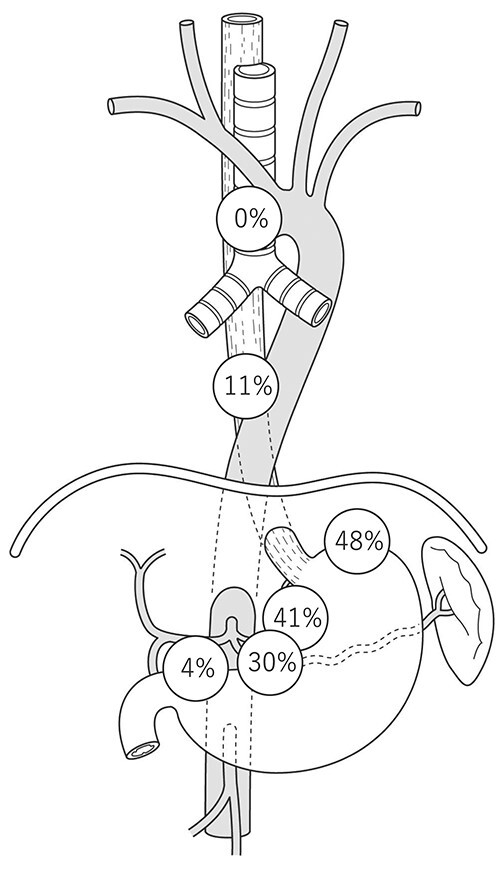
Distribution of metastatic LNs in pT1N+ AC of the EGC. (AC, adenocarcinoma; EGC, esophagogastric junction; LN, lymph node.)

A sentinel LN (SLN) is defined as the first LN on the direct lymphatic drainage pathway from a primary tumor site. We previously reported the SLN mapping of EC.^10^ In this study, radio-guided detection was used to identify SLN. A total of 75 patients with ESCC who were diagnosed preoperatively with T1N0M0 or T2N0M0 primary EC were enrolled. SLNs were identified successfully in 71 (95%) of 75 patients. The distribution of the identified SLNs was widely spread from the cervical to abdominal areas. Especially in ESCC located at the midthoracic esophagus, the rate of SLNs at the right supraclavicular LN was greater than 15%, and the rate of SLNs at the left gastric artery was ⁓11%, which indicated that the lymphatic drainage route of the esophagus was originally widely spread, even without LN metastasis (Fig. 1). The technical feasibility of SLN biopsy for EAC and ESCC was recently reported, which would aid in the development of SLN mapping for EGJ cancer.^11^^,^^12^ The distribution of LN metastasis in early stage disease reflects the variety of lymphatic routes shown in the SLN study. Akutsu *et al*. previously evaluated the sites and frequencies of the overall and initial LN metastases of patients with clinical T1N0 EC who were enrolled in a four-arm prospective study (JCOG0502) that compared esophagectomy with chemoradiotherapy for clinical T1N0 EC in both randomized and patient-preference arms.^13^ In middle thoracic cases, LN metastasis was observed in the neck, mediastinal, and abdominal regions, and pathologic SLN spread to all three fields (Fig. 2). Gertler *et al.*^11^ reported the prevalence of LN metastasis in pT1N+ cancer at the esophagogastric junction (EGJ). As shown in Figure 3, the distribution was localized around the EGJ at least in pT1 tumors located at the EGJ.^14^

### Location of the primary tumor and distribution of LN metastasis

The distribution of SLN and LN metastasis describes the complexity of lymphatic flow. As the disease progresses, LN metastasis spreads more widely, even in the curative stage. Therefore, to develop an optimal treatment strategy for EC, the location of the LN metastasis should be evaluated within all of the surgically resectable stages. In line with T1 cancer, the location of the primary tumor is the main contributor to the evaluation of the spread of metastatic LNs.

Hagens *et al*. conducted a systematic review of 14 articles to identify the distribution pattern of metastatic LN in relation to histology, tumor location, and T stage in 8952 patients with EC.^15^ Of the patients, 8543 (95%) had ESCC and 409 (5%) had EAC. Although the classification of the LN location differed among the studies, for patients with an upper thoracic tumor, LN metastases were frequently seen along the right recurrent nerve (60%), followed by cervical paraesophageal LNs (right 34%, left 22%). For patients with a middle thoracic tumor, the prevalence of LN metastases was highest along the right recurrent nerve (23%), right cervical paraesophageal LNs (24%), and middle thoracic paraesophageal LNs (23%). In patients with a tumor in the lower thoracic esophagus, the highest prevalence of LN metastases was along the left gastric artery (28%) and lower thoracic esophagus (23%). Furtheremore, metastatic lesions were found in both the mediastinal and abdominal LN stations, even after neoadjuvant treatment such as chemoradiotherapy.^16^

When investigating the distribution of LN metastasis in EGJ cancer, the lymphadenectomy field of the affected patients must be evaluated. Without upper mediastinal and cervical LN resection, the incidence of LN metastasis around those fields cannot be assessed. Kurokawa *et al.* conducted a prospective nationwide multicenter study in patients with cT2–T4 adenocarcinoma (AC) or squamous cell carcinoma (SCC) located within 2.0 cm of the EGJ. Patients were enrolled before surgery, and prespecified LNs were dissected by either the abdominal transhiatal or right transthoracic approach.^17^ A total of 371 patients were enrolled, among whom 358 underwent surgical resection. Most of the LNs with a high incidence of metastasis (>10%) were perigastric LNs with a node around the left gastric artery, whereas the rate of LN metastasis at the lower mediastinum was <5%. However, when the authors focused on patients with ≥2 cm esophageal invasion, ˃10% of patients were observed to have lower mediastinal LN metastasis. Furthermore, in those patients with either AC with esophageal involvement of >3.0 cm or SCC, the incidence rate of right recurrent laryngeal nerve LN metastasis was ⁓5%. Although we need to wait for the survival analysis to evaluate the efficacy of LN dissection for each station, the study accurately elucidated the distribution of LN metastases of EGJ cancer.

### Histology and LN metastatic patterns

One of the major debates is whether histology is important in assessing LN metastatic patterns. Needless to say, the location of the primary tumor, which is the primary contributor to LN metastatic patterns, differs greatly between ESCC and EAC. To conduct a fair assessment, it would be appropriate to concentrate on EGJ cancer, which has an exact number of patients in both ESCC and EAC. However, even when the anatomical tumor locations were the same, upper and middle mediastinal node dissection was omitted more frequently in AC patients than in SCC patients in clinical practice, resulting in an unavoidable limitation when assessing the prognostic impact of mediastinal LN dissection. As a result, it would be worthwhile to evaluate it in a prospective study in which the range of LN dissection is defined in the protocol on the location of the primary tumor. The tumor histology and distribution of LN metastasis did not show a significant correlation in the aforementioned Japanese prospective nationwide multicenter study of patients with EGJ cancer.^17^ As a result, the recommended field of lymphadenectomy for EGJ cancer is determined based on the length of esophageal invasion without taking histology into account. The representative studies on LN metastasis patterns in EC were summarized in Table 2.

**Table 2:** Representative studies on lymph node metastasis patterns in EC

Author (year)	Study layout	Country	Histology	Number of study participants	Details
Takeuchi (2009)	Prospective interventional study	Japan	SCC	75	The distribution of SLN in cT1-2N0M0 was described
Akutsu (2016)	Observational study using prospectively collected data	Japan	SCC	211	In cT1N0, the distribution of overall and initial LN metastases was assessed
Gertler (2014)	Retrospective research	Germany	SCC, AC, GC	793	Evaluated the prevalence and localization of LN metastasis in pT1
Hagens (2020)	Systematic review	NA	SCC, AC	8952	Described the prevalence of LN metastases per histologic subtype and primary tumor location
Kurokawa (2019)	Prospective interventional study	Japan	SCC, AC	371	Evaluated the optimal extent of LN dissection for EGJ tumors

SCC, squamous cell carcinoma; AC, adenocarcinoma; LN, lymph node; GC, gastric cancer; EGJ, esophagogastric junction; SLN, sentinel LN; EC, esophageal cancer.

### Presence of LN around thoracic duct

One of the subcategories of regional LNs is the LN around the thoracic duct (TD). Udagawa *et al.* previously reported the presence of LN around TD (TDLN) in the adipose tissue surrounding the TD running between the thoracic esophagus and the descending aorta^18^ and its metastatic rate. The metastatic incidence was 2.2% in pT1b/T2, whereas it was 10.0% in pT3/T4. Subsequently, we investigated the distribution of TDLN metastasis. In that study, TDLN was subdivided into TDLN Ut/Mt/Lt based on its location.^19^ The mean TDLN Ut/Mt/Lt numbers were 0.89/0.56/0.44. Furthermore, 11% of patients who underwent TD resection displayed TDLN metastasis. Metastatic TDLNs were observed on the same or cranial level of the primary lesion in most patients with TDLN metastasis. Because this trend was consistent in our follow-up study with an expanded cohort and longer follow-up duration,^20^ those results suggest that the lymphatic route along the TD, which runs from the caudal to the cranial side, seeds the cancer cells to the TDLN. The presence of TDLN was also confirmed in a cadaver study, which discovered TDLN in six of seven cadavers (86%), with a median number of 1 (range, 0–6).^21^

## SURGICAL APPROACH TO ERADICATING A WIDE RANGE OF LN METASTASES AND ITS EFFICACY

Because of the abundant lymphatic routes in the submucosal layer of the esophagus and its anatomical location, LN metastases can spread widely, even in the early stage. To eradicate tumors in the regional field, a strategy for extended LN dissection has been established, namely, three-field LN dissection (3FD).^22^ In esophagectomy, 3FD is defined as a procedure for cervico-thoraco-abdominal LN dissection. In the cervix, supraclavicular LN and paracervical esophageal nodes must be dissected. In a thoracic lymphadenectomy, LNs around the bilateral recurrent laryngeal nerve, paraesophageal LN, paratracheal LN, posterior mediastinal LN, and supradiaphragmatic LN are included in routine dissection. In the abdominal field, paracardial LN, LNs along the lesser curvature, LNs along the trunk of the left gastric artery, LNs around the abdominal esophagus, and infradiaphragmatic LNs are dissected. Although only a few randomized studies have been conducted on this topic,^23^ a large number of retrospective studies have reported the survival advantage of 3FD, and systematic reviews have supported positive results indicating that 3FD could show a favorable prognosis.^24^^,^^25^

However, an extended LN dissection might increase the incidence of postoperative complications. To evaluate the efficacy of LN dissection for each LN station, the efficacy index (EI), which is estimated by multiplying the incidence of metastasis and the 5 year overall survival rate of patients with LN metastasis for each station, was used.^26^ Tachimori *et al*. reviewed ⁓3800 ESCC patients using nationwide registry data established by the JES.^27^ In this study, the EI of the cervical node was relatively high in tumors located at the upper or midthoracic esophagus and intermediate in lower thoracic tumors. Regarding the abdominal nodes, the EI was high in lower thoracic tumors and decreased as the tumor location became higher. Therefore, it was concluded that the range of LN dissection should be selected based on the location of the primary tumor.

Li *et al.* are currently conducting a randomized phase III trial to evaluate the superiority of esophagectomy with 3FD for ESCC. They reported the short-term outcomes in 2020.^28^ Four hundred patients were randomized to either 3FD or two-field LN dissection. The authors reported that the rate and severity of postoperative complications were comparable between the two groups.

How LN metastasis affects prognosis after neoadjuvant chemotherapy (NAC) differs across stations. It was reported that clinically evaluated downstaging during neoadjuvant chemoradiotherapy (NACRT) is a prognostic indicator in terms of the correlation between response to neoadjuvant treatment and prognosis. Recently, Hagen’s *et al*., published a systematic review and meta-analysis that found that patients with LN downstaging had a survival benefit, but pathological response evaluation in LN did not become a significant prognostic marker.^29^ The efficacy of lymphadenectomy would differ depending on the perioperative therapy and its response.^30^^,^^31^ Thus, a larger study focusing on patients with or without neoadjuvant therapy would be more useful in developing an individualized treatment strategy for EC.

Although TDLN is usually included in regional LNs, the survival impact of dissecting TDLNs remains controversial. In our previous study that evaluated the long-term outcomes in patients who had TDLN metastasis, the recurrence-free survival and overall survival of patients with TDLN metastasis were almost identical to those with positive LN metastasis in extraregional LNs such as supraclavicular LNs.^20^ Ohkura *et al.* investigated the EI of TDLN. Their results showed that in ECC patients with cT3–4, there was no statistically significant difference in EI between TDLNs and non-TDLNs.^32^ Therefore, they concluded that TDLN resection was at least as effective as the dissection of other regional LNs. Conversely, several studies have insisted that TD resection provided a minimal contribution to improving survival. Oshikiri *et al.* analyzed 12,237 patients from JES registry data. The 5 year overall survival and cause-specific survival rates were comparable between the TD-resected and preserved group, without significant differences. Because no prospective comparative study has been conducted to evaluate the efficacy of TD resection with TDLN dissection, it would be challenging to conclude whether TDLN resection contributes to survival.

## CURRENT ADVANCEMENT OF MULTIDISCIPLINARY TREATMENT

Although surgical resection has been a mainstay for surgically resectable EC, the efficacy of a single modality is not satisfactory. To further improve oncologic outcomes, multidisciplinary treatment has been established worldwide as a standard treatment for EC and consists of surgery, chemotherapy, and radiotherapy.^33–35^ The overview of pivotal studies for multidisciplinary treatment for EC were described in Table 3. NAC followed by surgery for surgically resectable stages is primarily performed in Japan and the United Kingdom,^7^^,^^8^^,^^36^ whereas NACRT is provided in the majority of Western societies.^37^

**Table 3:** Overview of pivotal studies in terms of multidisciplinary treatment for EC

Author (year)	Treatment	Histology	*n*	OS (%)	*P*
Cunningham (2006)	ECF + Surgery + ECF	AC	250	36 (5 years)	*P* = 0.009
	Surgery alone		253	23 (5 years)	
van Hagen (2012)	Paclitaxel/Carboplatin/41.4Gy + Surgery	AC/SCC	175	59 (5 years)	*P* = 0.011
	Surgery alone		188	48 (5 years)	
Ando (2012)	5-FU/Cisplatin + Surgery	SCC	166	55 (5 years)	*P* = 0.04
	Surgery +5-FU/Cisplatin		164	43 (5 years)	
Al-Batran (2019)	FLOT + Surgery + FLOT	AC	356	45 (5 years)	*P* = 0.012
	ECF/ECX + Surgery + ECF/ECX		360	36 (5 years)	
Kelly (2021)	Paclitaxel/Carboplatin/41.4Gy + Surgery + Nivolumab	AC/SCC	532	22.4 months (MST of DFS)	*P* < 0.001
	Paclitaxel/Carboplatin/41.4Gy + Surgery + Placebo		260	11.0 months (MST of DFS)	
Kato (2022)	5-FU/Cisplatin + Surgery	SCC	185	62.6 (3 years)	
	5-FU/Cisplatin/Docetaxel + Surgery		183	72.1 (3 years)	*P* = 0.006
	5-FU/Cisplatin/40.4Gy + Surgery		178	68.3 (3 years)	ns

OS, overall survival; ECF, Epirubisin+Cisplatin+5-FU; AC, adenocarcinoma; SCC, squamous cell carcinoma; FLOT, 5-FU + Oxaliplatin+Docetaxel; ECX, Epirubisin+Cisplatin+Capexytabin; MST, median survival time; DFS, disease-free survival; ns, not significant; EC, esophageal cancer.

The Japan Clinical Oncology Group (JCOG) conducted a multicenter phase III trial comparing the efficacy of neoadjuvant cisplatin and 5-FU (CF) therapy with adjuvant CF therapy for ESCC.^38^ Consequently, the survival advantage of neoadjuvant CF was confirmed. To further improve the outcome for NAC in the treatment of ESCC, JCOG has been conducting a three-arm phase III trial, JCOG1109, which assesses the superiority of docetaxel, cisplatin, and 5-FU (DCF) over CF and the superiority of chemoradiotherapy with CF over CF as preoperative therapy.^39^ The result of the primary endpoint was reported, which demonstrated significantly improved overall survival in the group receiving neoadjuvant DCF when compared with the CF group, whereas NACRT did not. Subsequently, we conducted a nationwide retrospective study using real-world data from 85 Japanese esophageal centers and proved that neoadjuvant DCF showed a remarkable survival advantage over CF in patients with surgically resectable advanced ESCC.^40^ Therefore, neoadjuvant DCF therapy is recognized as a standard treatment.

In Europe, the Dutch CROSS trial proved the efficacy of NACRT using carboplatin and paclitaxel.^37^ Especially for ESCC, the pathological complete response was detected in 49% of participants. The German FLOT4 trial reported the efficacy of triplet chemotherapy using fluorouracil, oxaliplatin, and docetaxel (FLOT) compared with epirubicin, cisplatin, and fluorouracil or epirubicin, cisplatin, and capecitabine therapy.^41^ Thus, as a result of several well-designed epoch-making clinical trials from various esophageal societies, there has been an improvement in multidisciplinary treatments.

Given that neoadjuvant therapy has become the standard treatment, adding adjuvant therapy can be considered to eliminate residual diseases after surgery. As a tolerable option after gastrointestinal cancer surgery, nivolumab, an immune checkpoint inhibitor, was proven to extend disease-free survival as adjuvant therapy in patients with EC who received NACRT followed by esophagectomy in the CheckMate 577 trial.^42^ Patients with stage II/III esophageal or gastroesophageal junction cancer who had received NACRT and underwent esophagectomy were randomized to receive either adjuvant nivolumab or placebo. Consequently, the median duration of disease-free survival was 22.4 months in the nivolumab group as compared with 11.0 months in the placebo group (hazard ratio = 0.69; *P* < 0.001). Nomura *et al.* conducted phase II study PIECE trial in which adjuvant S-1 monotherapy, as another option, was provided for a half-year after esophagectomy.^43^ Adjuvant S-1 therapy showed acceptable toxicities and a promising prognosis. Overall, although there is a need to reevaluate the survival advantage of adjuvant therapy, it could be an ideal treatment strategy for those who have a high risk of postoperative recurrence.

**Fig. 4:**
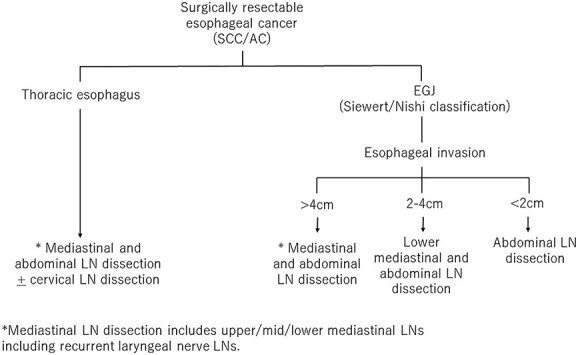
Surgical strategy based on the location of the primary tumor.

The recommended field of lymphadenectomy can be determined based on the location of the primary tumor and the LN metastasis patterns of EC. Regardless of histology, upper/mid/lower mediastinal LN dissection, including recurrent laryngeal nerve LNs, recommended when a tumor exists in the thoracic esophagus. When a tumor is found at the EGJ, the recommended field of lymphadenectomy is based on the length of esophageal invasion. This information was incorporated into the flow chart shown in Figure 4. The recommended perioperative treatment, on the contrary, could be diverse and would be chosen based on the patient’s background, clinicopathological factors, and standard treatment for each country.

## CONCLUSION

Due to the abundant lymphatic flow and the anatomical location of the esophagus, the distribution of LN metastasis of EC can spread widely from the cervical to the abdominal field. Their locations are closely linked to the location of the primary tumors. Alternatively, therapeutic efficacy must be evaluated based on prognosis and risk of lymphadenectomy. To improve the oncologic outcomes of patients with EC, the ideal combination of various multidisciplinary treatment approaches and extensive LN dissection must be established.

## CONFLICT OF INTEREST

Dr Kitagawa has received grants and personal fees from ASAHI KASEI PHARMA CORPORATION, grants, personal fees and other from ONO PHARMACEUTICAL CO., LTD., grants and personal fees from Otsuka Pharmaceutical Factory, Inc., grants and personal fees from Nippon Covidien Inc., grants, personal fees and other from TAIHO PHARMACEUTICAL CO., LTD, grants, personal fees and other from CHUGAI PHARMACEUTICAL CO., LTD., grants and personal fees from KAKEN PHARMACEUTICAL CO., LTD., personal fees from AstraZeneca K.K., personal fees from Ethicon Inc., personal fees from Olympus Corporation, personal fees from SHIONOGI & CO., LTD., personal fees and other from Bristol-Myers Squibb K.K., personal fees from MSD K.K., personal fees from Smith & Nephew KK, personal fees from ASKA Pharmaceutical Co., Ltd., personal fees from MIYARISAN PHARMACEUTICAL CO. LTD., personal fees from Toray Industries, Inc., personal fees from DAIICHI SANKYO COMPANY, LIMITED, personal fees from Chugai Foundation for Innovative Drug Discovery Science, personal fees from Nippon Kayaku Co., Ltd., grants from Yakult Honsha Co. Ltd., grants from Otsuka Pharmaceutical Co., Ltd., grants from TSUMURA & CO., grants from Sumitomo Pharma Co., Ltd., grants and personal fees from EA Pharma Co., Ltd., grants from Eisai Co., Ltd., grants from Kyowa Kirin Co., Ltd., grants from MEDICON INC., grants from Takeda Pharmaceutical Co., Ltd., grants from TEIJIN PHARMA LIMITED., personal fees from Intuitive Surgical G.K., outside the submitted work.
